# Tocotrienols Modulate a Life or Death Decision in Cancers

**DOI:** 10.3390/ijms20020372

**Published:** 2019-01-16

**Authors:** Shiau-Ying Tham, Hwei-San Loh, Chun-Wai Mai, Ju-Yen Fu

**Affiliations:** 1 School of Biosciences, Faculty of Science and Engineering, University of Nottingham Malaysia Campus, Jalan Broga, Semenyih 43500, Malaysia; ShiauYing.Tham@nottingham.edu.my; 2 Biotechnology Research Centre, University of Nottingham Malaysia Campus, Jalan Broga, Semenyih 43500, Malaysia; 3 School of Pharmacy, International Medical University, Bukit Jalil, Kuala Lumpur 57000, Malaysia; chunwai_mai@imu.edu.my; 4 Centre for Cancer and Stem Cell Research, Institute for Research, Development and Innovation, International Medical University, Bukit Jalil, Kuala Lumpur 57000, Malaysia; 5 Nutrition Unit, Product Development and Advisory Services Division, Malaysian Palm Oil Board, 6 Persiaran Institusi, Bandar Baru Bangi, Kajang 43000, Malaysia

**Keywords:** tocotrienols, cancer, cell death, cell survival, autophagy, apoptosis

## Abstract

Malignancy often arises from sophisticated defects in the intricate molecular mechanisms of cells, rendering a complicated molecular ground to effectively target cancers. Resistance toward cell death and enhancement of cell survival are the common adaptations in cancer due to its infinite proliferative capacity. Existing cancer treatment strategies that target a single molecular pathway or cancer hallmark fail to fully resolve the problem. Hence, multitargeted anticancer agents that can concurrently target cell death and survival pathways are seen as a promising alternative to treat cancer. Tocotrienols, a minor constituent of the vitamin E family that have previously been reported to induce various cell death mechanisms and target several key survival pathways, could be an effective anticancer agent. This review puts forward the potential application of tocotrienols as an anticancer treatment from a perspective of influencing the life or death decision of cancer cells. The cell death mechanisms elicited by tocotrienols, particularly apoptosis and autophagy, are highlighted. The influences of several cell survival signaling pathways in shaping cancer cell death, particularly NF-κB, PI3K/Akt, MAPK, and Wnt, are also reviewed. This review may stimulate further mechanistic researches and foster clinical applications of tocotrienols via rational drug designs.

## 1. Introduction

Cancer incidence rate has been increasing steadily every year, imposing significant social and economic impacts globally. In 2018, it was estimated that new cancer cases and mortality would increase to 18.1 million and 9.6 million, respectively, for the world population. In other words, 1 in 5 men and 1 in 6 women may develop cancer in their lifetime, while 1 in 8 men and 1 in 11 women may die from cancer [1]. In the United States (US) alone, it was predicted that there would be about 1.74 million new cancer cases, and approximately 600,000 people would eventually die from this disease in 2018. The estimated national expenditure for cancer care in the US was $147.3 billion in 2017. This figure is expected to increase continuously due to a high prevalence of cancer cases in aged population [2].

Chemotherapy has been the pillar for cancer disease management. However, the effectiveness of these drugs is often limited by challenges such as tumor immune evasion [3,4], high-dose toxicities, and drug resistance development through tumor microenvironment [4] or inappropriate cancer metabolism [5], which eventually lead to cancer metastasis and recurrence. Hence, more effective and potent anticancer agents are needed to save more lives from cancer death. Guided by several previous successes of isolating chemotherapeutic agents from plant sources, such as etoposide, paclitaxel, and vinblastine, considerable attention has been given to identify more effective anticancer agents or novel chemical entities from plant reservoirs [6,7,8,9,10,11,12]. Exhibiting cancer selectivity and exerting multitargeted actions for effectively killing various types of cancers, tocotrienols (T3) appear as a promising, novel anticancer candidate. The anticancer mechanisms of tocotrienols, including antiproliferation, promotion of apoptosis, and inhibitions of angiogenesis, cancer invasion, and metastasis, have been previously well reported and reviewed [13,14,15]. In fact, these pleiotropic effects portray value-added actions of tocotrienols in targeting cancers.

Tocotrienols, which belong to the vitamin E family, are naturally occurring compounds that can be found in plant seeds, such as rice bran, oil palm, and annatto [16]. They exist in four isomeric forms, namely, alpha (α)-, beta (β)-, gamma (γ)-, and delta (δ)-T3, which are distinguished by the position and number of methyl groups in the chromanal head. Depending on the source of tocotrienols, their composition of T3 isomers varies. For example, γ-T3 is predominantly higher in rice and palm compared to annatto, which mainly consists of δ-T3. Of note, γ-T3 and δ-T3 have been reported to exert most potent anticancer effects [17,18,19,20]. Other than pure isomers, tocotrienol-rich fraction (TRF) is the most commonly available form of vitamin E oral supplement, containing a mixture of α-, γ-, δ-T3, and some α-tocopherol. TRF has also been shown to exhibit apoptosis [21] and induce cell cycle arrest [22] and growth inhibition of human colon xenografts in nude mice [23]. Hence, the anticancer effects of γ-T3, δ-T3, and TRF are emphasized in this review.

Therapeutic intervention targeting a single hallmark of cancer de facto is unlikely to cure the disease [24]. This is because cancer arises from multiple mutations and lies within an aberrant and complex physiological network, rendering a unique identity for each tumor in different individuals [25]. In the process of transforming healthy cells to cancer cells, the core changes include increased proliferation and decreased cell death [26]. Therefore, it is important to reverse the state when applying an anticancer therapy. Particularly, targeting cell death and survival pathways may offer advantages for rapid and effective elimination of cancer cells and avoid drug resistance development and subsequent progression of cancers into advanced stages.

Several recent reviews have comprehensively summarized the general biological effects of tocotrienols, including anti-inflammation, antihyperlipidemic, immunomodulation, neuroprotection, gastroprotection, hepatoprotection, nephroprotection, radioprotection, and anticancer [27,28,29]. Despite the enormous pharmacological and biological potentials of tocotrienols, as reviewed previously [15,27,28,29], the actions of tocotrienols in promoting cell death and blocking survival via various pathways have not been reviewed in an integrated manner. Hence, the present review comprehensively gathers the evidences of different cell death modalities induced by tocotrienols in order to provide a global view on the molecular mechanisms and an integrated understanding between cell death and survival signaling pathways for therapeutic benefits.

## 2. Programmed Cell Death in Cancer

**Figure 1:**
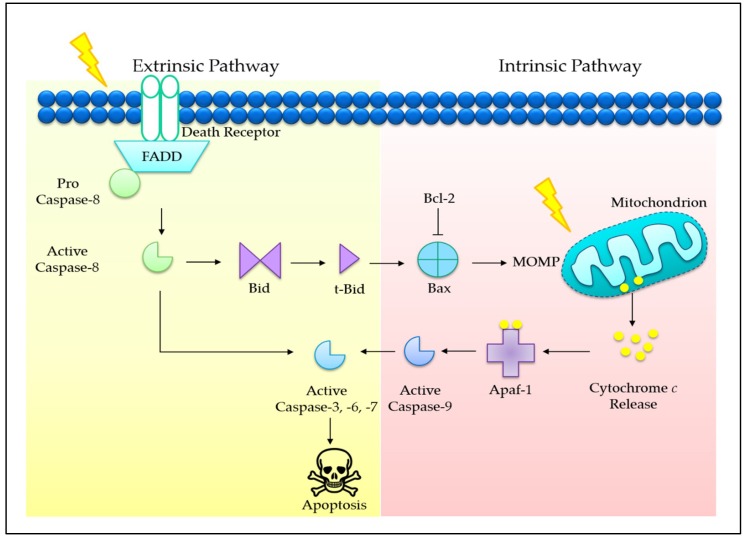
Extrinsic and intrinsic pathways of apoptosis. The extrinsic pathway begins outside of a cell and involves the binding of extracellular death ligands to their respective transmembrane death receptors. The activation of tumor necrosis factor (TNF) receptor superfamily, such as first apoptosis signal (Fas), takes place, leading to activation of caspase-8 (initiator caspase) and recruitment of the Fas-associated protein with death domain (FADD) adapter molecule. It transduces a downstream signaling cascade to the effector caspase (caspase-8), leading to the proteolytic activation of caspase-3. In fact, the extrinsic pathway can cause amplification of cascade via intrinsic mitochondrial pathway whereby caspase-8 cleaves Bid to promote mitochondrial outer membrane permeabilization (MOMP) [32]. The intrinsic pathway is initiated by internal stimuli, such as genetic damage, growth factor deprivation, hypoxia, oxidative stress, and flux of calcium ions (Ca^2+^) [33]. The stimuli then perceive cell death signals via the mitochondrion, which represents the metabolic status of a cell. The MOMP is often regarded as the primary step required for activation of caspases. Proapoptotic and antiapoptotic B-cell lymphoma 2 (Bcl-2) family proteins are involved in regulating the permeability of outer mitochondrial membrane [34,35]. Upon apoptotic stimulus, MOMP takes place, leading to the release of cytochrome *c* from the intermembrane space. In the cytoplasm, cytochrome *c* engages apoptotic protease activating factor 1 (Apaf-1) and eventually leads to the activation of caspase-9 (initiator caspase). Following that, caspase-9 activates executioner caspases, such as caspase-3, -6, and -7, which subsequently cause the downstream biochemical events, leading to apoptosis [36].

**Figure 2:**
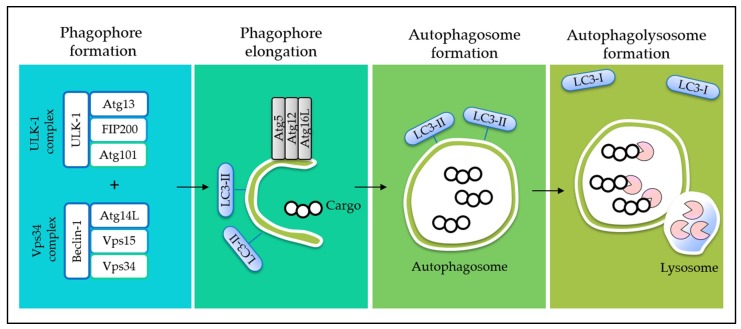
The process of autophagy. During autophagy, phagophore (cup-shaped, double-membrane sac) emerges in cytoplasm, driven by unc-51-like kinase 1 (ULK1) complex and vacuolar protein sorting (Vps) 34 complex. The expansion of phagophore is facilitated by Atg5–12/Atg16L complex to uptake cargos from the cytoplasm into a double-membrane autophagosome. The loaded autophagosome then fuses with lysosome to allow the degradation of cargo by lysosomal proteases, while microtubule-associated protein light chain 3 (LC3-I) will be recycled back to cytosol. The endogenous LC3-I, present in the cytoplasm, is processed to LC3-II and bound to the autophagosome during autophagy. Therefore, the ratio of LC3-I (water soluble) and LC3-II (lipidated) is often used as a marker to assess autophagy. Then, the lysosomal permeases and transporters export amino acids and other by-products of degradation back to the cytoplasm, where they can be reused for building macromolecules and for metabolism [37]. Abbreviations: Atg, autophagy-related protein; FIP200, focal adhesion kinase family interacting protein of 200 kDa.

Programmed cell death (PCD) is an evolutionary conserved cellular mechanism, which is important during embryonic development, tissue morphogenesis, and for the removal of unwanted or damaged cells in our body [30]. There are two common types of PCD, namely, apoptosis (Type I) (Figure 1) and autophagy (Type II) (Figure 2). In addition, it has recently been proposed that necrosis, which is more popularly known as an accidental cell death, could also be a form of PCD, known as necroptosis (Type III PCD). Dysregulation of these pathways is often captured during cancer development, rendering enhanced cell survival in cancer cells [31]. Therefore, therapeutics that are able to provoke these mechanisms stand a chance to eliminate cancer cell population. Herein, evidences on the effects of tocotrienols toward PCD, particularly type I and type II PCD, are presented and discussed to provide a general landscape of different cell death modalities induced by tocotrienols.

## 3. Tocotrienols Act as a Potent Apoptosis Inducer

Targeting apoptotic pathways remains an attractive approach to effectively eliminate cancer cells without causing inflammation. For many years, tocotrienols have been gaining immense research attention due to their proapoptotic effect in various types of cancers, as previously reported in breast [38,39], lung [40], colon [23,41,42], brain [20,43], liver [44,45], cervix [46], blood [47], and skin [17,48] cancers. Various apoptotic mechanisms triggered by tocotrienols are presented in this section.

### 3.1. Tocotrienols Induce Mitochondria-Mediated Apoptosis

Mitochondria are tiny organelles in a cell, which exert both vital and lethal functions. In addition to serving as a powerhouse for fueling energy to cells, this organelle also contains homicidal molecules that can subject a cell to death [49]. Tocotrienols exhibit mitochondrial disruption capacity via mitochondrial outer membrane permeabilization (MOMP) induction [50,51,52], culminating in mitochondria-mediated apoptosis. In fact, MOMP is a critical event in the intrinsic apoptotic pathway. It has been reported that the blockade of mitochondrial permeability transition pore (MPTP) with cyclosporine A completely abolished the cytotoxic effects of TRF, β-T3, γ-T3, and δ-T3 in activated rat pancreatic stellate cells, which could support the growth and invasiveness of pancreatic ductal adenocarcinoma [53,54].

**Figure 3:**
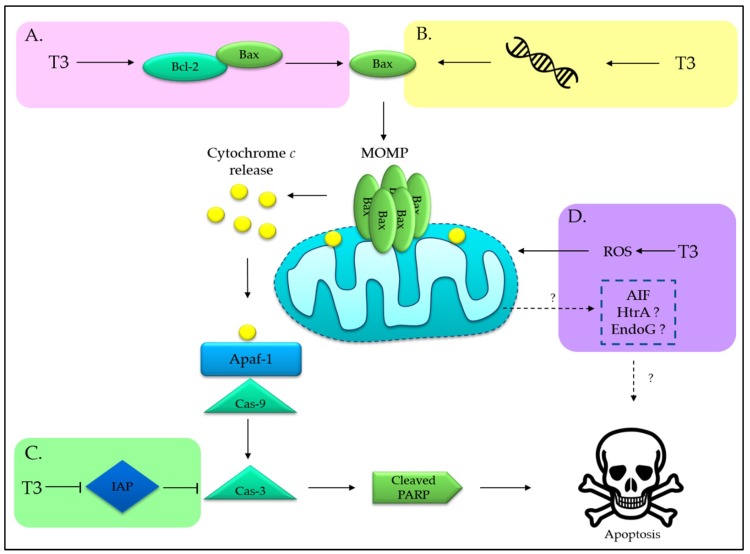
Proposed actions of tocotrienols (T3) in inducing mitochondrial pathway of apoptosis. A: direct displacement of Bcl-2 molecule by acting as a BH3 mimetic; B: transcriptional regulation of *Bax* gene expression; C: inhibition of IAP family; D: induction of caspase-independent apoptotic pathway after mitochondrial damage. Abbreviations: Apaf-1, apoptotic protease activating factor 1; Bcl-2, B-cell lymphoma 2; Cas-, caspase-; MOMP, mitochondrial outer membrane permeabilization; IAP, inhibitor of apoptosis protein; PARP, poly(ADP-ribose) polymerase; ROS, reactive oxidative species; AIF, apoptosis inducing factor; HtrA, high temperature requirement A; EndoG, endonuclease G. “?” indicates more investigations are required.

Although the actual role of tocotrienols in mitochondria-mediated apoptosis remains elusive, four potential interactions have been proposed (Figure 3). Several lines of evidence have reported that tocotrienols alter Bcl-2/Bax ratio, rendering depolarization of mitochondria [50,55,56]. A study conducted on neuroblastoma SH-SY5Y cells shed a light on the potential interaction between γ-T3 and B-cell lymphoma 2 (Bcl-2) proteins. This research showed that γ-T3 competes with 8-Anilino-1-naphthalenesulfonic acid ammonium salt (ANS) for binding to the hydrophobic groove of Bcl-2. Hence, it was suggested that γ-T3 acts as Bcl-2 homology 3 (BH3) mimetic to displace proapoptotic members from Bcl-2 sequestration. As a result, proapoptotic molecules become available to permeabilize the outer mitochondrial membrane and release cytochrome *c* to the cytosol, leading to caspase-9- and caspase-3-dependent apoptosis [57]. However, it will be more worthwhile if γ-T3, which is claimed to serve as an inhibitor of antiapoptotic Bcl-2 members, can be further characterized to allow development of derivatives that embrace a greater therapeutic efficacy [57].

On the other hand, another study revealed that the elevated Bax expression induced by δ-T3 correlates to the induction of zinc finger transcription factor, known as early growth response protein 1 (EGR1). This transcription factor binds to the *Bax* gene promoter, leading to the induction of proapoptotic Bax in pancreatic cancer cells [58].

Suppression of inhibitor of apoptosis (IAP) could be another target of tocotrienols in mitochondrial pathway. IAP can block apoptosis by deactivating caspases activity. Interestingly, tocotrienols have been reported to be able to downregulate the IAP level, such as cIAP-1, cIAP2, and survivin in colorectal cancer [59,60]. Of note, cancer displays an elevated IAP profile to promote uncontrollable cell division, which may hinder the effectiveness of treatments that rely on caspase activation. Hence, the ability to block the activity of IAP could be an opportunity to culminate in a complete activation of apoptosis.

Other than indirectly modulating the Bcl-2 family to induce MOMP, γ-T3-treated MDA-MB-231 breast cancer cells have been found to show a direct disruption of mitochondrial membrane potential independent from Bax/Bcl-2 ratio alteration and poly(ADP-ribose) polymerase (PARP) cleavage [50]. The authors suggested that cytochrome *c* release is not a critical event for apoptosis induction in this scenario. In another study, γ-T3 treatment on human T cell lymphoma Jurkat cells elevated mitochondrial reactive oxidative species (ROS) production [61]. Hence, it is plausible to suggest that γ-T3 could promote mitochondrial dysfunction, probably via ROS generation, leading to metabolic catastrophe and cell death, rather than engaging caspase-dependent intrinsic apoptosis. Additionally, it can also be speculated that γ-T3 induces caspase-independent cell death following the release of soluble intermembrane mitochondrial proteins, such as apoptosis inducing factor (AIF) [50], high temperature requirement A (HtrA), and endonuclease G (EndoG).

### 3.2. Tocotrienols Induce Endoplasmic Reticulum Stress

**Figure 4:**
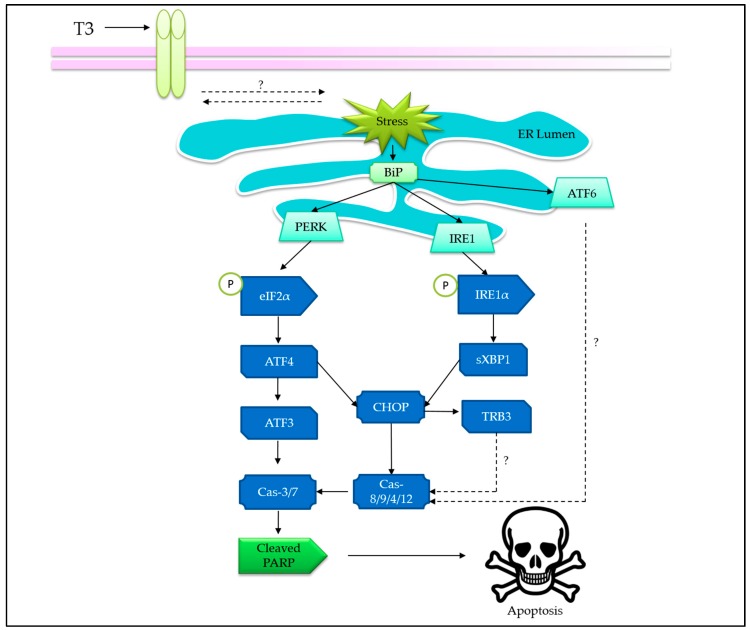
Overview of tocotrienols-induced ER-stress-mediated apoptosis. Tocotrienols interact with unknown receptor on cell surface to trigger ER stress. BiP dissociates from unfolded protein response (UPR) sensors (i.e., PERK, IRE1, and ATF6) to trigger the respective signaling pathways, leading to apoptosis. Abbreviations: BiP or GRP78, glucose-regulated protein; CHOP, CCAAT-enhancer-binding protein homologous protein; PERK, PKR-like ER-localized eIF2α kinase; IRE1, inositol-requiring enzyme 1; ATF, activating transcription factor; eIF2-α, eukaryotic initiation factor 2 alpha; Cas-, caspase-; PARP, poly(ADP-ribose) polymerase; sXBP1, spliced X-box binding protein 1, TRB3, tribbles 3. “?” indicates more investigations are required.

Endoplasmic reticulum (ER) experiences stress during perturbations in Ca^2+^ homeostasis, redox imbalance, altered protein glycosylation, or protein folding defects, leading to accumulation of unfolded or misfolded proteins in the ER lumen [62,63]. Eventually, the highly conserved unfolded protein response (UPR) pathway is triggered to alleviate the potentially toxic stress and restore the homeostasis [64]. A persistent and overwhelming ER stress will lead to apoptosis. Several studies have reported that tocotrienols exert ER stress as one of the mechanisms to induce apoptosis [65,66,67]. In this section, the evidences of tocotrienols-induced ER stress are gathered and aligned to the present understanding on apoptosis that is led by ER stress (Figure 4).

The involvement of ER-stress-mediated apoptosis, as triggered by tocotrienols, was first reported by Wali and co-workers [68]. Their work showed that γ-T3 activated protein kinase-like endoplasmic reticulum kinase (PERK) arm of the UPR in neoplastic mouse +SA mammary epithelial cells by phosphorylating (inhibition) eukaryotic translational initiation factor (eIF2α), leading to the increase in activating transcription factor 4 (ATF-4) [68]. Following that, the ER-stress-inducible nuclear protein, CCAAT-enhancer-binding protein homologous protein (CHOP), and tribbles 3 (TRB3) expressions were also increased. Knockdown of CHOP by siRNAs attenuated PARP cleavage induced by γ-T3, suggesting a CHOP-dependent, ER-stress-mediated apoptosis.

Another study conducted on γ-T3-treated MCF-7 and MDA-MB-231 breast cancer cells demonstrated an involvement of ER stress via both the PERK and inositol-requiring enzyme 1 (IRE1) pathways [69], leading to remarkable increment in their downstream targets, such as activating transcription factor 3 (ATF3) and CHOP, in response to γ-T3 treatment [69]. The resultant apoptosis was evident by caspase-7 activation and PARP cleavage. In addition, ATF3 knockdown using siRNA abrogated the apoptosis, suggesting that ATF3 is a putative molecular target in γ-T3-induced apoptosis [69].

In HeLa cervical cancer cell line, γ-T3 and δ-T3 induces ER stress via the activation of IRE1 pathway, which in turn mediates the alternative splicing of X-box binding protein 1 (XBP-1) mRNA and modulates CHOP transcription [70], leading to subsequent activation of caspase-9, -8, and -12 [70]. In an attempt to study the underlying molecular mechanism, a specific estrogen receptor inhibitor (fulvestrant) employed as a competitive inhibitor to tocotrienols showed a reduction in the expression of proapoptotic genes [70]. Hence, it was hypothesized that γ-T3 may activate the “orphan” receptor (that also binds to fulvestrant), leading to IRE-1 activation and XBP-1 splicing, which finally induce apoptosis that is cognate with the activation of ER stress.

To our knowledge, the involvement of ATF6 pathway was reported as null in at least two studies [69,71]. Differing from PERK and IRE pathways, the activation of ATF6 requires translocation and cleavage in Golgi apparatus, which is often associated with enhanced survival in cancer cells [69,72]. It is possible that tocotrienols-induced ER stress is rapid and favors apoptosis, leaving behind the prosurvival option. Although a reduction of ATF6α level has been observed in +SA cells, the spatial information in relation to its cleavage has not been studied [68]. Possibly, the ATF6 level reduction is due to cell death but not cleavage in the Golgi apparatus. Hence, the involvement of ATF6 pathway in tocotrienols-induced ER-stress-mediated apoptosis requires further investigation.

The activation of extrinsic apoptotic pathway being coupled to ER stress suggests a close connection between them. For instance, it has been reported that both death receptor 5 and CHOP were upregulated upon treatment of γ-T3 on MDA-MB-231 and MCF-7 breast cancer cells [73]. However, the sequence of activation and the dominance of the respective pathways remain elusive.

### 3.3. Co-Involvement of Endoplasmic Reticulum Stress and Mitochondria-Mediated Apoptosis

The interconnectivity between organelles could play an important role in perceiving stress upon receiving cancer drugs and influences the life or death decision in the cancer cells. In fact, increasing evidences have shown that ER stress may cooperate with mitochondria for stress signal amplification, thus culminating in apoptosis [74]. For instance, mitochondria-mediated apoptosis was evident in δ-T3-treated BLM and A375 human melanoma cell lines by cytochrome *c* release and augmentation of Bax/Bcl-2 ratio. At the same time, ER-stress-related proteins, such as PERK, p-eIF2α, ATF4, CHOP, IRE1α, and caspase-4, were activated. The application of salubrinal (inhibitor of ER stress) has been found to successfully block the cytotoxicity of δ-T3, signifying the importance of ER stress for apoptosis induction [71]. In fact, ATF/CHOP or IRE-1 ER stress sensors could be the mediators to connect ER stress to mitochondrial pathway of apoptosis [75], potentially via the modulation of Bcl-2 family [76]. It is interesting to note that several studies have suggested ATF4 may facilitate the transcriptional upregulation of BH3-only proteins, such as p53 upregulating the modulator of apoptosis (PUMA) and NOXA during ER stress [77,78,79]. On the other hand, CHOP also downregulates Bcl-2 protein, thus incurring higher susceptibility to MOMP [77]. Additionally, it appears that calcium signaling could play an important role in the ER–mitochondrial communication [76]. It has been suggested that the elevated flux of Ca^2+^ from ER, subsequently leading to cytochrome *c* release, could happen independent of MPTP [77]. Thus far, only limited studies have been conducted on understanding the connectivity of tocotrienols-induced ER stress and mitochondria-mediated apoptosis. Time course studies on molecular targets for both pathways can be conducted in order to elucidate the superiority of each pathway in tocotrienols-induced apoptosis, which may offer a better understanding on the mechanism of action and pave an avenue for discovering novel therapeutics for cancers.

### 3.4. Co-Involvement of Extrinsic and Intrinsic Pathways

In addition to tocotrienols inducing apoptosis via intrinsic mitochondrial pathway, a few studies have shown that both intrinsic and extrinsic pathways are activated concurrently. Although death-receptor-mediated extrinsic pathway and mitochondria-mediated intrinsic pathway are triggered by different stimuli, the two pathways are converged on executioner caspases (e.g., caspase-3, -7). γ-T3 has been reported to engage both intrinsic and extrinsic pathways in Hep3B human hepatoma cells, as demonstrated by elevated activities of caspase-8, -9, -3 and accompanied by upregulation of truncated Bid and Bax, resulting in PARP cleavage [45]. In addition, co-elicitation of intrinsic pathway by inducing Ca^2+^ release, loss of mitochondrial membrane potential, and increase in Bax/Bcl-2 ratio, and extrinsic pathway via upregulating surface expression of Fas and FasL in γ-T3-treated human T-cell lymphoma (Jurkat cells) has been reported. Eventually, apoptosis was induced via activation of caspase-8, -9, and -3 and PARP cleavage [61]. Moreover, the involvement of Bid, Bax, cytochrome *c*, disruption of mitochondrial membrane permeabilization-dependent caspase-8 activation induced by α-T3, γ-T3, and δ-T3 have also been reported in A549 lung adenocarcinoma and U87MG brain glioblastoma [40]. In addition, activation of both intrinsic and extrinsic pathways has been reported in leukemic cells, involving upregulation of genes in Bcl-2, caspases, and death receptor families [80]. These evidences therefore suggest a convergence of extrinsic and intrinsic pathways at mitochondria, which potentially serve as an apoptosis signal amplification center. The cooperative interaction by simultaneous activation of intrinsic and extrinsic pathways can potentially lead to enhanced cell death through functional complementation as mutation of more than one pathway is common in cancer [65,81]. However, the benefit of this cooperative action induced by tocotrienols may be more remarkable in type II cancer cells, which requires mitochondrial pathway for death-receptor-induced apoptosis [82].

## 4. Interplay between Autophagy and Apoptosis

Autophagy has been well known as a mechanism for cell survival under immense cell stress, whereas apoptosis is a pro-death decision when a cell experiences an unrecoverable damage. Therefore, inhibition of autophagy is often seen as an approach to promote apoptosis. For instance, a study showed that application of autophagy inhibitor 3-methyladenine (3-MA) potentiated apoptosis induced by dietary tocotrienols in breast cancer cells [83], suggesting an antagonistic role of autophagy to apoptosis.

**Figure 5:**
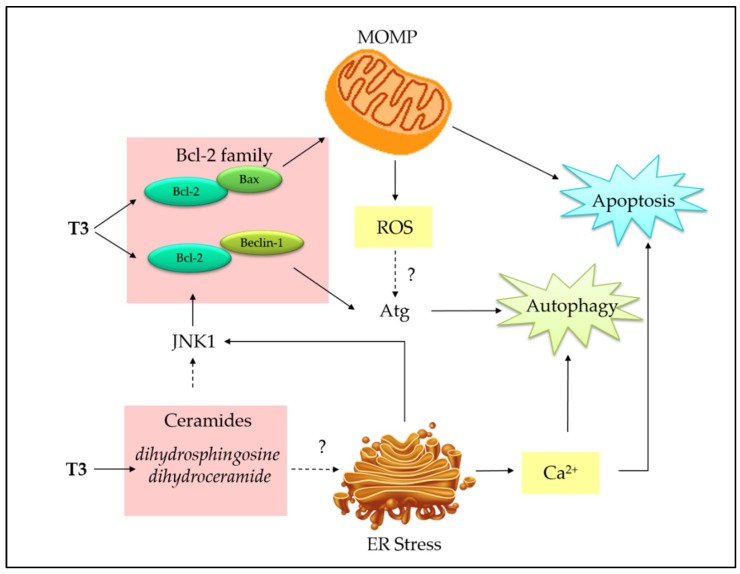
Schematic representation of potential interactions between tocotrienols-induced apoptosis and autophagy. Tocotrienols bind to Bcl-2 protein to displace proapoptotic protein (i.e., Bax) and proautophagic protein (Beclin-1) to initiate apoptosis and autophagy, respectively. Bax initiates MOMP to induce apoptosis, leading to generation of ROS, which serves as a second messenger to trigger the expression of autophagy-related protein (Atg). Tocotrienols upregulate de novo synthesis of ceramides, which can activate JNK signaling by phosphorylating Bcl-2 family proteins and induce Ca^2+^ release by ER stress, eventually resulting in apoptosis and autophagy. “?” indicates more investigations are required.

On the contrary, accumulating evidences have shown that autophagy and apoptosis act together to induce cell death in cancer. However, the connections at molecular level are multifaceted and poorly understood [84]. We attempt to establish a connection between tocotrienols-induced apoptosis and autophagy based on currently available literature (Figure 5). Mitochondria and ER are proposed as the target organelles to drive the connection between the two pathways.

The activated pancreatic stellate cells treated with TRF exhibit the ability of tocotrienols to co-induce mitochondria-mediated apoptosis and autophagy [53]. In the study, TRF was reported to cause apoptosis, as indicated by depolarized mitochondrial membrane, cytochrome *c* release, increased DNA fragmentation, and caspase activation. Concurrently, autophagy was also apparent by the formation of autophagic vacuoles and LC3-II accumulation. An attempt to block apoptosis with zVAD-fmk caspase inhibitor showed a converse enhancement in autophagy, suggesting autophagy could serve as a backup plan to execute the cells when apoptosis fails. In this case, autophagy is engaged in an immediate and active process, rather than as a passive “stress adaptive” function. It has been proposed that tocotrienols interconnect apoptosis and autophagy at mitochondria because the blockade of MPTP with cyclosporine A completely abolishes apoptosis and autophagic response [85].

The actual mechanism has not been fully elucidated; however, it has been suggested that tocotrienols may initiate intrinsic mitochondrial pathway by displacing proapoptotic (Bax, Bak, BH3-only proteins) and proautophagic (Beclin-1) proteins from BH3 docking sites of Bcl-2 or Bcl-xL proteins, resulting in both apoptosis and autophagy [85]. In addition, ROS generated from compromised mitochondria by TRF treatment has been proposed to connect two pathways because ROS is essential to activate autophagic enzymes, such as Atg4 [85], as well as to amplify apoptotic signal. Although this study was not developed from a cancer model, the dual pathways activation shed a light on the novel function of tocotrienols. Later, concurrent activation of apoptosis and autophagy was correspondingly reported in other cancer cell lines, such as breast and prostate [86,87].

In γ-T3-treated mouse (+SA) mammary tumor cells and human (MCF-7 and MDA-MD-231) breast cancer cell lines, proautophagic proteins, such as LC3-II/LC3-I ratio and Beclin-1 levels, and the corresponding increased levels of apoptotic markers including Bax/Bcl-2 ratio, cleaved caspase-3, and cleaved PARP, demonstrated the concurrent induction of autophagy and apoptosis [88]. In the following study, the authors showed autophagy inhibitor surrogating the cytotoxicity [89], further confirming the presence of cytotoxic autophagic cell death. In prostate cancer cells, γ-T3 induced elevated intracellular ceramides (dihydrosphingosine and dihydroceramide), which appear to be potent mediators of apoptosis and autophagy [87]. Ceramides have been well reported as a key player in apoptosis by inducing both intrinsic and extrinsic apoptotic pathways [90]. In comparison, the connection of ceramides with autophagy is emerging; the ceramides may activate c-Jun N-terminal 1 (JNK1) to phosphorylate Bcl-2 (inactivation), eventually leading to the dissociation of Beclin-1 from Bcl-2 [90].

In addition, γ-T3 can simultaneously activate ER-stress-mediated apoptosis and autophagy to promote cell death in MCF-7 and MDA-MB-231 breast cancer cells [89]. Autophagy markers, such as Beclin-1, LC3-II, lysosomal-associated membrane protein 1 (LAMP-1), and cathepsin-D, were upregulated in both breast cancer cell lines. Concurrently, ER stress apoptotic markers, such as phospho-PERK, phospho-eIF2α, BiP, IRE1α, ATF-4, CHOP, and TRB3, were upregulated, suggesting an involvement of ER-stress-mediated apoptosis and autophagy induction [89]. Based on recent evidences, it can be speculated that ER stress induces Ca^2+^ release into cytosol, which could serve as a second messenger to co-activate apoptosis and autophagy [89,90,91]. Potentially, ER-stress-induced IRE1α stimulates JNK and p38 signaling, leading to inactivation of Bcl-2, as mentioned in the previous paragraph.

## 5. Tocotrienols Target Prosurvival Signaling Pathways

**Figure 6:**
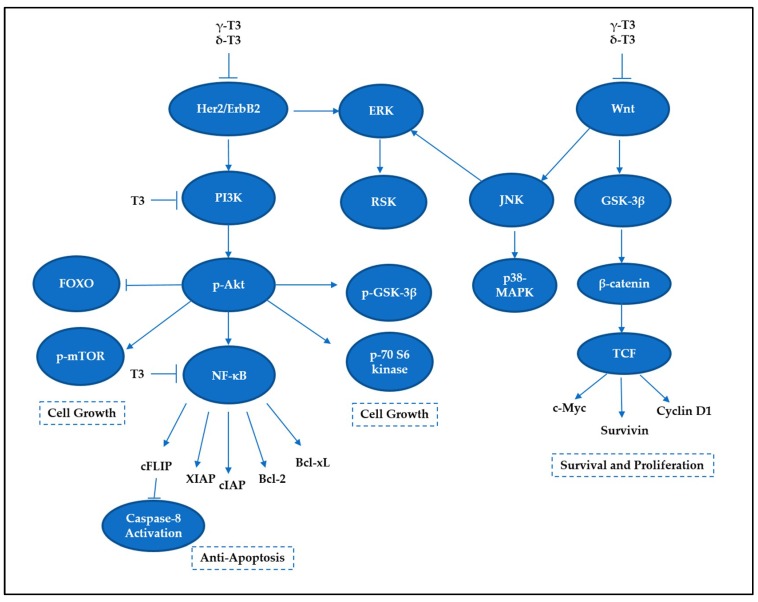
Tocotrienols suppress cell survival signaling pathways in cancers.

Targeting only the cell death pathways may not be sufficient to eliminate cancer cells. Cell death signaling can be overcome by stimulation of cell survival via alternative modes, leading to a more complicated problem, such as development of drug-resistant cancer cells. Tocotrienols have been previously reported to concurrently activate cell death programs and downregulate prosurvival signaling pathways, such as nuclear factor kappa B (NF-κB), phosphoinositide 3-kinase (PI3K)/Akt, Wingless and INT-1 (Wnt), and mitogen-activated protein kinases (MAPKs) (Figure 6). Therefore, the following section describes the capability of tocotrienols in targeting cell survival and deliberating implications in the cell death mechanisms.

### 5.1. NF-κB Family

The NF-κB activity is crucial in modulating survival-promoting proteins, which would lead to poor responses toward anticancer therapies. Inhibition of NF-κB by tocotrienols has been reported to reduce the expression of various proteins linked to cell survival, such as antiapoptosis (e.g., Bcl-2, Bcl-xL, cFLIP, IAP-1, IAP-2, XIAP, Bfl-1/A1, TRAF1, and survivin), proliferation (e.g., c-Myc and cyclin D1), inflammation (COX-2, cytokines), invasion (e.g., MMP-9, ICAM-1, ELAM-1, and VCAM-1), and neoangiogenesis (e.g., VEGF) [92,93,94]. Tocotrienols have been shown to reduce constitutive NF-κB activity in mammary epithelial [95], prostate cancer [19], multiple myeloma [92], lung adenocarcinoma [96], oral cancer [97], and colon carcinoma [56] cell lines. A study conducted on pancreatic cancer showed that γ-T3 was able to suppress NF-κB activity in vitro and in vivo [98]. Correspondingly, δ-T3 significantly affected NF-κB DNA binding activity in pancreatic cancer cells [99]. This inhibitory activity appears to be exclusive for γ-T3 and δ-T3 isomers as α-T3 and β-T3 had no significant effect on NF-κB activity [99]. It has been proposed that γ-T3 and δ-T3 block the phosphorylation and degradation of IκB (inhibitor of NF-κB), leading to inhibition of IκB kinase (IKK) complex activation as well as suppression of nuclear translocation of p65 [18,92,99]. Another in vivo study combining γ-T3 with capecitabine also demonstrated a significant inhibition in NF-κB and NF-κB-regulated proteins, such as cyclin D1, COX-2, MMP-9, ICAM-1, Bcl-xL, survivin, and XIAP, in gastric tumor tissues [100]. As a result, the blocking of NF-κB pathway leads to the suppression of antiapoptotic gene products and potentiation of apoptosis [92]. Considering that apoptotic products are closely related to NF-κB activity, agents that can reduce the NF-κB level could serve as a potential “multitasking agent”.

### 5.2. PI3K/Akt Signaling

The PI3K pathway plays a determining role in regulating cell survival. The activation of Akt phosphorylates and inhibits the proapoptotic Bcl-2 family members, such as Bad [101]. The antiproliferative effect of γ-T3 in neoplastic +SA mouse mammary epithelial cells is mediated by a reduction in PI3K/PDK-1/Akt mitogenic signaling [102]. Suppression in this signaling pathway further leads to death-receptor-independent caspase-8 activation and reduction of intracellular FLIP expression [103,104]. In pancreatic cancer cell lines (MIA PaCa-2 and Panc-28), γ-T3 and δ-T3 could effectively prevent Akt activation. A further study revealed that several downstream targets of Akt were affected by γ-T3, such as mTOR, S6 kinase (Ser 240/244), GSK-3β, and FOXO3 [105]. These results collectively suggest that the two tocotrienol isoforms can induce apoptosis in pancreatic cancer cells through the suppression of vital cell survival and proliferative signaling pathways [105].

### 5.3. MAP Kinase Signaling

MAPK signaling pathway plays a critical role in the outcome of, and sensitivity to anticancer therapies [106]. There are three important MAPK members for maintenance of cells, namely, extracellular signal-regulated kinases (ERKs), c-Jun N-terminal kinases (JNKs), and p38-MAPKs. ERKs have been found to be important for cell survival, whereas JNKs are deemed stress-responsive and thus involved in apoptosis [107]. On the other hand, p38-MAPK has been reported to exhibit a dual role as cell death and cell survival regulators depending on the types of cell and stimulus received [108]. Of note, it has been proposed that the roles of p38 and JNK regulate the balance of apoptosis and autophagy in response to chemotherapeutic agents, portraying a significant role of MAPK signaling in deciding life or death matter.

γ-T3 and δ-T3 treatments in pancreatic cells have been found to show reductions of ERK activation and its downstream mediator RSK (ribosomal protein S6 kinase), which correlate to the downregulation of HER/ErbB2 expression [105]. On the other hand, a research illustrated that γ-T3 treatment caused suppressions of ERK and p38 MAPK but an activation of ERK in T-cell lymphoma, leading to both intrinsic and extrinsic apoptosis [61]. In contrast, another study reported that γ-T3 elevated death receptors, DR4, and DR5, which are regulated through ERK activation [60]. In fact, the contradictory roles of ERK activation for promoting cell death or cell survival could be delineated by studying the subcellular localization of ERK proteins [109]. For instance, translocation of ERK1/2 to nucleus carries an anticancer role, while positioning to mitochondria may promote cell survival [110]. Collectively, these studies reveal a connection between proliferative signaling and apoptosis, which further strengthens the versatility of tocotrienols in targeting cancers.

### 5.4. Wnt Signaling

Wnt signaling is one of the key cascades for regulating development and stemness; overactivation of this pathway commonly occurs in cancer, predominantly in colorectal cancer [111]. Wnt pathway is commonly divided into canonical (β-catenin-dependent) and noncanonical (β-catenin-independent) signaling [111]. Serving as an important driver in the canonical pathway, the activation of Wnt receptors leads to translocation of β-catenin into nucleus. Subsequently, it forms an active complex with lymphoid enhancer factor (LEF), T-cell factor (TCF), and histone-modifying coactivators to initiate transcriptional activities for multiple processes such as cell proliferation and cell survival, such as c-Myc, cyclin D1, and survivin [111,112]. Additional evidence has shown that activation of Wnt signaling can overcome the apoptosis mediated by Notch in gastric cancer [113], portraying the importance of Wnt signaling in shaping apoptotic cell death.

δ-T3 has been reported to inhibit SW620 colon cancer cells by downregulating the expression levels of Wnt-1, β-catenin, c-Jun, and cyclin D1 in the Wnt signal pathway [114]. In addition, γ-T3 inhibited cell viability through suppression of β-catenin/TCF signaling in human colon carcinoma HT-29 cells [42]. TRF suppressed the growth of human colon cancer xenografts in Balb/c nude mice via the Wnt pathway, by which the expressions of β-catenin, Wnt, and c-Myc proteins in xenografts were significantly downregulated [23]. In breast cancer cells, γ-T3 reversed the epithelial-to-mesenchymal transition in human breast cancer via the inhibition of canonical Wnt signaling [115]. Taken together, the ability of tocotrienols in targeting Wnt pathway suggests a value-added therapy to effectively regulate proliferation, apoptosis, and metastasis simultaneously.

## 6. Current and Future Perspectives of Tocotrienols

To date, undoubtedly, promising anticancer effects and molecular targets of tocotrienols in promoting cell death and suppressing survival in cancers have been revealed from numerous in vitro (Table 1) and in vivo (Table 2) studies. These valuable preclinical findings have expectedly warranted further investigations for clinical applications, some recent examples of which are summarized in Table 3. Despite ample evidence of the therapeutic benefits of tocotrienols in various types of cancers, current clinical studies have only embarked on breast, colon, pancreas, lung, and ovary cancers. Other cancer types, such as brain, blood, gastric, and prostate cancers, with positive therapeutic potentials may be lining up in forthcoming clinical trials.

Yet, poor oral bioavailability remains the main hurdle limiting the in vivo therapeutic efficacy of tocotrienols. Saturable uptake in the transport mechanism within intestine and bloodstream, leading to low bioavailability, has therefore greatly compromised the efficacy and potency of tocotrienols via oral intake [116]. Several formulation strategies have been found to enhance the oral absorption of tocotrienols by at least 3 folds, including self-emulsifying delivery systems [117] and nanostructured lipid carriers [118]. In addition, alternative routes of administration have been investigated to circumvent the limitations associated with oral absorption. One such approach is the application of nanoformulation that enables tocotrienols to be administered via intravenous and topical routes. Polymer-conjugated tocotrienols [119] and entrapment of tocotrienols in nanovesicles [120] have been investigated for intravenous injection, while tocotrienol nanoemulsions have been investigated for topical applications [121]. These formulations markedly improved the antiproliferative activities of tocotrienols in vitro and tumor suppression properties in vivo. Hence, further studies on clinical translation of these novel tocotrienol formulations are warranted.

It has been widely reported that administration of high-dose tocotrienols is a futile maneuver due to high metabolic degradation in vivo [43]. Prominently, the hypomethylated forms of tocotrienols, i.e., δ-T3 and γ-T3, show the highest cellular metabolism. In fact, high metabolism of T3s in vivo are associated with the induction of drug metabolizing enzymes, such as cytochrome P450 enzyme (CYP450) and glucuronosyltransferase 1A1 (UGT1A1) as well as the induction of multidrug resistance protein-1 (MDR1) via pregame-X-receptor (PXR) and steroid and xenobiotic receptor (SXR). These enzymes appear to deliver positive cytotoxic effects to cancer cells; however, coadministration of high-dose tocotrienols with other drugs may potentially interfere the metabolism, thereby affecting the therapeutic efficacy of these drugs [122,123,124]. Hence, a synergistic combination of tocotrienols with other anticancer agents at low doses can augment the therapeutic efficacy and potency (both bioactives) as well as reduce the dose-limiting toxicities (i.e., chemotherapeutic agents). In addition, a combined treatment approach could reduce the risk of developing drug resistance in cancer cells. As “one drug one target”-based targeted therapy (e.g., tyrosine kinase inhibitors) is inclined to drug resistance [125], combinatorial application of tocotrienols that concurrently targets multiple signaling pathways can effectively eradicate cancer cell population [126]. In fact, the multitargeted anticancer actions offered by tocotrienols are regarded as a valuable feature that could potentially pave an avenue into polypharmacology, which advocates “one drug multiple targets” [127]. Furthermore, combining existing drugs with tocotrienols could also serve as an immediate and cost-effective solution. So far, combination therapies using tocotrienols with traditional chemotherapeutics, plant bioactives, and targeted inhibitors have shown positive therapeutic responses in vitro and in vivo, as reviewed by Eitsuka et al. [128]. However, metabolism, toxicities, and pharmacokinetics studies of combined treatments in vivo are still lacking, more investigations are therefore necessary to advance effective drug combinations toward a tangible clinical application in the near future.

At present, two completed clinical trials have demonstrated positive benefits of tocotrienols in cancer management. Although statistically insignificant due to limited sample size, the first clinical study conducted on breast cancer patients showed that TRF in combination with tamoxifen improved breast-cancer-specific survival compared to the tamoxifen group (NCT01157026) [38]. The second clinical trial conducted on pancreatic ductal neoplasia patients reported that δ-T3 was well tolerated by patients, while the tumor samples showed an elevated caspase-3 activity, suggesting an enhanced apoptosis due to δ-T3 intervention (NCT00985777). Furthermore, another clinical study investigating the new formulation of γ-δ-T3 showed an enhanced bioavailability in healthy subjects (NCT01571921), undoubtedly heralding a great milestone for accelerating its application for cancer therapy. Of note, a number of clinical trials are currently in progress to study the anticancer effects of tocotrienols and/or in combination with other therapeutic agents (Table 3). Specifically, interventions of tocotrienols are involved as neoadjuvant and adjuvant cancer treatments as well as health supplements [129]. Taken together, these studies could provide a better understanding on clinical applications of tocotrienols with optimal benefits.

While clinical trials of tocotrienols are important to identify the therapeutic benefits in various types of cancer, mechanistic studies should also be extensively conducted in order to explore further potentials upon acquiring the putative underlying mechanisms of action. In particular, the potential of tocotrienols in inducing nonapoptotic cell death has not been well described and certainly deserves more research attention. For instance, paraptosis induced by δ-T3 and γ-T3 colon cancer cells have shed a light on caspase-independent cell death [130,131]. The authors also highlighted the potential involvement of mitochondria and ER in tocotrienols-induced paraptosis, which is worthwhile to be investigated further [130]. In addition, the crosstalk between cell death signaling is important to provide an insight into the molecular mechanisms, which may be essential for striking a balance between cell survival and cell death as well as their role as targets for the development of novel therapeutic approaches [132]. Hence, considerable attention should be given to understand the tocotrienols-induced molecular crosstalk to gain insights on the cancer-specific mode of action. Moreover, as we are moving into an era of cancer molecular profiling, a comprehensive mechanism of action could better translate tocotrienols in clinical practice via rational drug designs.

## 7. Conclusions

Tocotrienols exhibit versatility in inducing cell death by modulating various mechanisms in cancers. Tocotrienols exhibit potent proapoptotic capacities by inducing mitochondria-mediated apoptosis and ER-stress-mediated apoptosis; this co-elicitation is believed to serve as an enhanced cancer-killing strategy. In fact, engagement of both extrinsic and intrinsic apoptotic pathways by tocotrienols could lead to a more effective cancer elimination, particularly in Type II cells, which require mitochondrial pathway in inducing apoptosis. Apart from that, proper modulation of cell survival signaling underscores a pivotal role in shaping the ultimate cancer cellular demise. Of note, autophagy appears to account for a better role in cell death than its classical association in “cell survival” to serve as a backup mechanism in tocotrienols-induced apoptosis. Overall, the pleiotropic effects of tocotrienols in inducing various cell death mechanisms while hampering the prosurvival pathways of cancer cells portray a value-added action in fighting cancers. Yet, the oral bioavailability and metabolic degradation associated with high dose have constrained the clinical applications of tocotrienols. Perhaps the ongoing advancements in nanoformulation and combined treatment approaches could outshine the current cancer regimens using tocotrienols to achieve better therapeutic outcomes.

## Figures and Tables

**Table 1:** Exemplar in vitro studies for elucidating the molecular targets of tocotrienols-induced cell death.

Cancer Type	Cell Line(s)	Molecular Target(s)	Reference(s)
Bladder	T24 5637 J82 UMUC-3	↑ p21, p27, Bax, caspase-3, cleaved PARP, SHP-1 ↓ cyclin D1, Bcl-2, Bcl-xL, Mcl-1, ETK phosphorylation, STAT3	[133]
Brain	U87MG	↑ caspase-8, Bid, cytochrome *c*, Bax	[41,134]
Breast	MDA-MB-231	↑ caspase-8, caspase-9, caspase-7, caspase-3, cleaved PARP, DR5, DR4, p-JNK, p-c-Jun, p-p38, BiP, ATF3, ATF4, p-PERK, p-IRE1α, p-eIF2α, CHOP, LC3-II/I, Beclin-1, Bax ↓ NF-κB, cyclin D1, cyclin D3, CDK4, Bcl-2, PI3K, p-AKT, p-mTOR, XIAP	[69,73,88,135,136]
	MCF-7	↑ caspase-8, caspase-9, caspase-7, caspase-3, Bax, cleaved PARP, ATF3, BiP, CHOP, p-PERK, p-IRE1α, p-EIF2α, ATF4, LC3-II/I, Beclin-1, DR5, p-JNK, p-c-Jun, p-p38, MIC-1, EGR-1, cathepsin D ↓ cyclin D1, cyclin D3, CDK4, NF-κB, Bcl-2, PI3K, p-AKT, p-mTOR	[69,70,73,88,136,137]
	+SA	↑ LC3-II/I, Beclin-1, Bax, cleaved PARP, cleaved caspase-3 ↓ Bcl-2, PI3K, p-AKT, p-mTOR	[88]
	SKBR3	↓ p-ERK1/2	[138]
	MDA-MB-435	↑ cleaved PARP, p-JNK-1, JNK1, p-c-Jun, c-Jun, TGFβRII, TGFβRI	[139]
Cervix	HeLa	↑ Bax, cytochrome *c*, caspase-12, caspase-9, caspase-8, caspase-3, IL-6, XBP-1, p-IRE-1α, BiP, CHOP, XBP-1, cleaved PARP ↓ PCNA, cyclin D3, p16, CDK6	[46,47,140,141]
	CaSki	↑ p53, Bax, caspase-3 ↓ MEK-2, ERK	[141,142]
Colon	HT29	↑ p21, Bax, caspase-9, caspase-3 ↓ Bcl-2, NF-κB p65, β-catenin, cyclin D1, c-Myc, survivin	[42,56,143,144]
	SW620	↓ Wnt-1, β-catenin, cyclin D1, c-Jun, MMP-7	[130,131]
	HCT116	↑ p21 ↓ cIAP-1, cIAP-2, survivin, cyclin D1, c-Myc, MMP-9, VEGF, ICAM-1, CXCR4, NF-κB	[59,144]
	DLD-1	↑ p21, p27, caspase-7, caspase-9 ↓ hTERT	[144,145,146]
Gastric	SGC-7901	↑ Bax, caspase-3, caspase-9, cleaved PARP ↓ Bcl-2, c-Myc, p-ERK1/2, Raf-1	[147,148]
	SNU-5	↓ NF-κB	[100]
	SNU-16	↑ cleaved PARP ↓ cyclin D1, Bcl-2, MMP-9, CXCR4, VEGF, NF-κB	[100]
Leukemia	ED40515	↑ caspase-3, caspase-6, caspase-7, caspase-9, PARP, Bcl-2, Bcl-xL, XIAP ↓ FDFT1, NF-κB	[47]
	HL-60	↑ cleaved Bid, cytochrome *c* release, caspase-8, caspase-9, caspase-3	[51]
	NB-4	↑ cleaved Bid, cytochrome *c* release, caspase-8, caspase-9, caspase-3	[51]
Lung	A549	↑ caspase-3, caspase-8, Bid, cytochrome *c*, Bax, cleaved PARP ↓ Notch-1, Hes-1, Bcl-2, NF-κB, uPA, survivin, Bcl-XL, MMP-9	[41,134,149,150]
	H520	↑ PARP, caspase-3 ↓ Notch-1, Hes-1, Bcl-2, NF-κB, survivin, Bcl-XL	[96,149]
	H1299	↓ Notch-1, Hes-1, uPA, MMP-9	[149]
Pancreas	MIA PaCa-2	↑ E-cadherin, EGR-1, Bax, p27Kip1 ↓ NF-κB, Bcl-2, cIAP-1, survivin, cyclin D1, c-Myc, COX-2, VEGF, MMP-9, ICAM-1, CXCR4, N-cadherin, vimentin, p-MEK, p-AKT, p-GSK-β	[58,98,151,152]
	L3.6pl	↑ E-cadherin ↓ N-cadherin, vimentin, VEGF, MMP-9	[151]
	BXPC3	↑ p27Kip1 ↓ p-MEK, p-AKT, p-ERK	[152]
	SW1990	↑ p27Kip1 ↓ p-MEK, p-AKT, p-ERK	[152]
	PANC-1	↑ p21	[153]
	Pancreatic cancer stem cell	↑ cleaved PARP ↓ Nanog, Sox-2, Oct-4, Notch-1, p-AKT, pERK	[151]
Prostate	PC-3	↑ caspase-9, cytochrome *c,* cleaved PARP, LC3-II ↓ p-Akt, β-catenin, Id-1, Bcl-2	[19,87]
	PC-3 (stem cell-like)	↑ caspase-3, cleaved PARP ↓ Id-1	[154]
	LNCaP	↑ caspase-9, caspase-8, caspase-7, caspase-3, cytochrome *c*, cleaved PARP, LC3-II ↓ Id-1, p-Akt	[19,87]
Skin	G361	↑ cleaved PARP, caspase-7, caspase-9, caspase-3, E-cadherin, β-catenin, γ-catenin ↓ Snail, vimentin, α-SME, Twist	[17]
	C32	↑ cleaved PARP, caspase-7, caspase-9, caspase-3, IκB, p-ATF2, p-c-Jun, p-SAPK/JNK ↓ PI3K p85, p-IKKα/β, IκBα/β, NF-κB p65, EGFR, Id-1, Id-3	[17]
	A375 (stem cell-like)	↓ ABCG2	[155]
	BLM	↑ caspase-3, caspase-4, cleaved PARP, Bax, BiP, PERK, p-eIF2α, IRE1α, ATF4, CHOP ↓ Bcl-2	[71]
	A375	↑ caspase-3, caspase-4, cleaved PARP, BiP, PERK, p-eIF2α, IRE1α, ATF4, CHOP, ERO1α ↓ Bcl-2, CDK4, Ras, caspase-3	[48,71]
	A2058	↓ CDK4, Ras, caspase-3	[48]
	B16	↑ p-ERK ↓ Tyrosinase, MC1R, MITF, TYRP-1, TYRP-2, p-p38	[156]

Note: ↑upregulation; ↓downregulation; p—, phosphorylated state. Abbreviations: ABCG2, ATP-binding cassette subfamily G member 2; Akt, or PKB, protein kinase B; ATF, activating transcription factor; ATP, adenosine triphosphate; Bcl-xL, B-cell lymphoma-extra large; BiP, or GRP78, glucose-regulated protein; CDK, cyclin-dependent kinase; CHOP, CCAAT-enhancer-binding protein homologous protein; cIAP, cellular inhibitor of apoptosis; COX-2, cyclooxygenase-2; CXCR4, C-X-C motif chemokine receptor 4; DR, death receptor; EGR, early growth response; eIF2-α, eukaryotic initiation factor 2 alpha; ERK1/2, extracellular signal-regulated protein kinases 1 and 2; ERO1α, endoplasmic reticulum oxidation 1; ETK1, epithelial and endothelial tyrosine kinase; FDFT1, farnesyl-diphosphate farnesyltransferase 1; GSK-β, glycogen synthase kinase 3 beta; Hes-1, hairy and enhancer of split-1; hTERT, human telomerase reverse transcriptase; ICAM-1, intercellular adhesion molecules-1; Id-1, inhibitor of differentiation/DNA binding; IκBα/β, inhibitor of kappa B alpha/beta; IKKα/β, IκB kinase alpha/beta; IL-6, interleukin 6; IRE-1, inositol-requiring enzyme 1; JNK, c-Jun N-terminal kinase; LC3, microtubule-associated protein 1A/1B-light chain 3; MC1R, melanocortin 1 receptor; Mcl-1, myeloid cell leukemia 1; MEK, mitogen-activated protein kinase; MIC-1, macrophage inhibitory cytokine 1; MITF, melanogenesis associated transcription factor; MMP, matrix metalloproteinases; mTOR, mammalian target of rapamycin; NF-κB, nuclear factor kappa B; Oct-4, octamer-binding transcription factor 4; p27Kip1, cyclin-dependent kinase inhibitor 1B; PARP, poly(ADP-ribose) polymerase; PCNA, proliferating cell nuclear antigen; PERK, PKR-like ER-localized eIF2α kinase; PI3K, phosphoinositide 3-kinase; ROS, reactive oxygen species; SAPK/JNK, stress-activated protein kinase/c-Jun NH2-terminal kinase; Sox-2, sex determining region Y-box 2; TGF-β1, transforming growth factor beta 1; TYRP, tyrosinase-related proteins; VEGF, vascular endothelial growth factor; uPA, urokinase-type plasminogen activator; Wnt, wingless/integrase; XBP, X-box binding protein; XIAP, X-linked inhibitor of apoptosis protein; α-SMA, alpha-smooth muscle actin.

**Table 2:** Exemplar in vivo studies of tocotrienols for various cancer treatments using mouse models.

Cancer Type	Tocotrienol(s)	Anticancer Effect(s)/Molecular Target(s)	Reference
Colon	TRF or δ-T3-enriched diet	δ-T3-enriched diet decreased the number of colorectal tumors in an animal model, but not in TRF-fed group. δ-T3-enriched diet suppressed COX-2 protein levels in colorectal mucosa.	[157]
Colon	TRF	TRF inhibited xenografts in mice by a regulation of Wnt pathways. TRF increased expression of Wnt pathways related factors, i.e., Axin-2, GSK-3β, APC and decreased the protein expression of Wnt-1, β-catenin and β-catenin target genes, i.e., *cyclin D1*, *c-Myc* and *survivin* in the xenografts.	[23]
Gastric	γ-T3	γ-T3 inhibited > 50% of tumor growth. γ-T3 downregulated microvessel density indicator CD31. γ-T3 downregulated NF-κB and NF-κB-regulated cyclin D1, COX-2, survivin, Bcl-xL, XIAP, ICAM-1, MMP-9 and VEGF.	[100]
Pancreas	γ-T3	γ-T3 inhibited cancer cell proliferation in tumor tissues. γ-T3 inhibited constitutive activation of NF-κB. γ-T3 significantly downregulated the expression of proinflammatory marker COX-2, suppressed the expression of invasion biomarker MMP-9, and inhibited the angiogenic biomarker VEGF in the tissues. γ-T3 reduced Bcl-2, cIAP-1, CXCR4, NF-κB, c-Myc.	[98]
Pancreas	δ-T3	δ-T3 inhibited pancreatic tumor growth and metastasis. δ-T3 inhibited epithelial-to-mesenchymal transition in pancreatic tumors as E-cadherin upregulated, N-cadherin, vimentin, VEGF, MMP-9, CD44 were downregulated. δ-T3 inhibited cancer cell proliferation and increased cleaved caspase-3.	[151]
Pancreas	δ-T3	δ-T3 inhibited cancer cell proliferation, decreased phosphorylated MAPK expression and induced expression of p27Kip1.	[152]
Pancreas	δ-T3	δ-T3 significantly enhanced the survival of mice. δ-T3 decreased levels of p-AKT, p-MEK, p-ERK, NF-κB and Bcl-xL and increased levels of p27Kip1, Bax, CK18 and activated caspase-3.	[158]
Prostate	γ-T3	γ-T3 inhibited tumorigenicity of PC-3 cells in mice.	[154]
Prostate	γ-T3	γ-T3 inhibited xenograft growth in nude mice.	[87]
Prostate	γ-T3	γ-T3 inhibited the growth of xenograft. γ-T3 reduced PCNA, Ki-67 and Id1 in tumor tissues. γ-T3 increased cleaved PARP and cleaved caspase-3. γ-T3 increased expression levels for the tumor suppressor gene (*E-cadherin*) and its repressor (Snail).	[159]
Skin	δ-T3	δ-T3 treatment disabled the formation of melanospheres completely in mice.	[155]
Skin	δ-T3	δ-T3 inhibited the growth and progression of melanoma xenografts in nude mice.	[71]

**Table 3:** Recent clinical trials conducted using tocotrienols for various cancer treatments.

Cancer Type	Target Application(s) of Tocotrienols	Drugs Involved	Phase: Status	ClinicalTrials.gov Identifier
Breast	Adjunct cancer treatment	TRF and Tamoxifen	Pilot trial: Completed in 2010	NCT01157026
Breast	Health supplement	Gamma-Delta Tocotrienols and TRF	1: Completed in 2013	NCT01571921
Breast	Neoadjuvant treatment	Epirubicin, Cyclophosphamide, Docetaxel, Paclitaxel, Trastuzumab, Pertuzumab and Tocotrienols	2: Ongoing	NCT02909751
Colon	Adjunct cancer treatment	Irinotecan, Oxaliplatin, Calcium Folinate, 5-Fluorouracil and Tocotrienols	2: Ongoing	NCT02705300
Lung	Adjunct cancer treatment	Cisplatin, Vinorelbine, Carboplatin and Tocotrienols	3: Ongoing	NCT02644252
Ovary	Adjunct cancer treatment	Bevacizumab and Tocotrienols	2: Ongoing	NCT02399592
Ovary	Cancer treatment	Cabazitaxel and/or Tocotrienols	2: Ongoing	NCT02560337
Pancreas	Cancer treatment	δ-T3	1: Completed in 2016	NCT00985777
Pancreas	Health supplement	δ-T3	1: Completed in 2016	NCT01450046
Pancreas	Health supplement	δ-T3	1: Completed in 2016	NCT01446952

Note: Examples of clinical studies that are registered at https://clinicaltrials.gov showing a status as accessed on 18th December 2018.

## Abbreviations

3-MA	3-Methyladenine
ABCG2	ATP-binding cassette subfamily G member 2
AIF	Apoptosis-inducing factor
Akt	PKB or protein kinase B
ANS	8-Anilino-1-naphthalenesulfonic acid ammonium salt
Apaf-1	Apoptotic protease activating factor 1
APC	Adenomatous polyposis coli
ATF4	Activating transcription factor 4
ATF6	Activating transcription factor 6
Atg	Autophagy-related protein
Bf1-1/A1	Bcl-2-related protein A1
BH3	Bcl-2 homology 3
BiP	GRP78, glucose-regulated protein
CDK	Cyclin-dependent kinase
CHOP	CCAAT-enhancer-binding protein homologous protein
cIAP	Cellular inhibitor of apoptosis
COX-2	Cyclooxygenase-2
CXCR4	C-X-C motif chemokine receptor 4
CYP450	Cytochrome P450 enzyme
DR	Death receptor
EGR1	Early growth response protein 1
eIF2-α	Eukaryotic initiation factor 2 alpha
ELAM-2	Endothelial cell adhesion molecule-2
EndoG	Endonuclease G
ER	Endoplasmic reticulum
ERK1/2	Extracellular signal-regulated protein kinases 1 and 2
ERO1α	Endoplasmic reticulum oxidation 1
ETK1	Epithelial and endothelial tyrosine kinase
FDFT1	Farnesyl-diphosphate farnesyltransferase 1
GSK-β	Glycogen synthase kinase 3 beta
Hes-1	Hairy and enhancer of split-1
hTERT	Human telomerase reverse transcriptase
HtrA	High temperature requirement A
IAP	Inhibitor of apoptosis protein
ICAM-1	Intercellular adhesion molecules-1
Id-1	Inhibitor of differentiation/DNA binding
IκB	Inhibitor of kappa B
IKKα/β	IκB kinase alpha/beta
IL-6	Interleukin 6
IRE-1	Inositol-requiring enzyme 1
JNK	c-Jun N-terminal kinase
LAMP-1	Lysosomal-associated membrane protein 1
LC3	Microtubule-associated protein 1A/1B-light chain 3
LRP	Low-density lipoprotein receptor-related protein
MAPK	Mitogen-activated protein kinase
MC1R	Melanocortin 1 receptor
Mcl-1	Myeloid cell leukemia 1
MDR1	Multidrug resistance protein-1
MEK	Mitogen-activated protein kinase
MIC-1	Macrophage inhibitory cytokine 1
MITF	Melanogenesis associated transcription factor
MMP	Matrix metalloproteinases
MOMP MPTP	Mitochondrial outer membrane permeabilization Mitochondrial permeability transition pore
mTOR	Mammalian target of rapamycin
NF-κB	Nuclear factor kappa B
Oct-4	Octamer-binding transcription factor 4
p27Kip1	Cyclin-dependent kinase inhibitor 1B
PARP	Poly(ADP-ribose) polymerase
PCD	Programmed cell death
PCNA	Proliferating cell nuclear antigen
PDK1	3-phosphoinositide-dependent protein kinase-1
PERK	Protein kinase R (PKR)-like endoplasmic reticulum kinase
PI3K	Phosphoinositide 3-kinase
PXR	Pregame-X-receptor
ROS	Reactive oxygen species
RSK	Ribosomal protein S6 kinase
SAPK/JNK	Stress-activated protein kinase/c-Jun NH2-terminal kinase
SHP-1	Src homology region 2 domain-containing phosphatase-1
Sox-2	Sex determining region Y-box 2
SXR	Steroid and xenobiotic receptor
TGF-β1	Transforming growth factor beta 1
TRA1	Transcription-associated protein 1
TRB3	Tribbles-related Protein 3
TYRP	Tyrosinase-related proteins
UGT1A1	Glucuronosyltransferase 1A1
ULK	Unc-51-like kinase
uPA	Urokinase-type plasminogen activator
UPR	Unfolded protein response
US	United States
VCAM-1	Vascular cell adhesion molecule-1
VEGF	Vascular endothelial growth factor
Vps	Vacuolar protein sorting
Wnt	Wingless/integrase
XBP	X-box binding protein
XIAP	X-linked inhibitor of apoptosis protein
